# Comparative genomics study reveals Red Sea *Bacillus* with characteristics associated with potential microbial cell factories (MCFs)

**DOI:** 10.1038/s41598-019-55726-2

**Published:** 2019-12-17

**Authors:** G. Othoum, S. Prigent, A. Derouiche, L. Shi, A. Bokhari, S. Alamoudi, S. Bougouffa, X. Gao, R. Hoehndorf, S. T. Arold, T. Gojobori, H. Hirt, F. F. Lafi, J. Nielsen, V. B. Bajic, I. Mijakovic, M. Essack

**Affiliations:** 1 Computational Bioscience Research Center (CBRC), King Abdullah University of Science and Technology (KAUST), Thuwal, 23955-6900 Kingdom of Saudi Arabia; 2 Department of Biology and Biological Engineering, Division of Systems & Synthetic Biology, Chalmers University of Technology, Kemivägen 10, 41296 Gothenburg, Sweden; 3 Biological and Environmental Sciences and Engineering Division (BESE), King Abdullah University of Science and Technology (KAUST), Thuwal, 23955-6900 Kingdom of Saudi Arabia; 4 Department of Biology, Science and Arts College, King Abdulaziz University, Rabigh, 21589 Kingdom of Saudi Arabia; 5 College of Natural and Health Sciences, Zayed University, 144534 Abu-Dhabi, United Arab Emirates; 6 Novo Nordisk Foundation Center for Biosustainability, Technical University of Denmark, 2800 Lyngby, Denmark; 7 Science for Life Laboratory, Royal Institute of Technology, Solna, Sweden

**Keywords:** Genome informatics, Bacterial genomics

## Abstract

Recent advancements in the use of microbial cells for scalable production of industrial enzymes encourage exploring new environments for efficient microbial cell factories (MCFs). Here, through a comparison study, ten newly sequenced *Bacillus* species, isolated from the Rabigh Harbor Lagoon on the Red Sea shoreline, were evaluated for their potential use as MCFs. Phylogenetic analysis of 40 representative genomes with phylogenetic relevance, including the ten Red Sea species, showed that the Red Sea species come from several colonization events and are not the result of a single colonization followed by speciation. Moreover, clustering reactions in reconstruct metabolic networks of these *Bacillus* species revealed that three metabolic clades do not fit the phylogenetic tree, a sign of convergent evolution of the metabolism of these species in response to special environmental adaptation. We further showed Red Sea strains *Bacillus paralicheniformis* (Bac48) and *B. halosaccharovorans* (Bac94) had twice as much secreted proteins than the model strain *B. subtilis* 168. Also, Bac94 was enriched with genes associated with the Tat and Sec protein secretion system and Bac48 has a hybrid PKS/NRPS cluster that is part of a horizontally transferred genomic region. These properties collectively hint towards the potential use of Red Sea *Bacillus* as efficient protein secreting microbial hosts, and that this characteristic of these strains may be a consequence of the unique ecological features of the isolation environment.

## Introduction

*Bacillus* species are ubiquitous Gram-positive bacteria known for their ability to survive in a wide variety of environments, including marine environments such as seawater^1^, tidal flat^2–4^, and sediments^5–10^; soil environments such as rhizospheres^11–15^; human gut samples^16–18^; as well as food samples such as dairy products^19,20^ and fermented soybeans^19^. For several *Bacillus* strains from such diverse environments, *in silico* methods^21,22^ and *in vitro* experiments^23–30^ have shown strong biosynthetic and superb protein secreting capabilities. This observation motivated using certain *Bacillus* as industrial producers for an array of pharmacologically and industrially relevant compounds including biosurfactants^31,32^, antimicrobials^33–35^, hydrolysis and deproteinization enzymes^36–38^, and livestock probiotics. The premise that species from different environments have unique biosynthetic capabilities is supported by a study that surveyed secondary metabolism gene clusters (SMGCs) in *Bacillus* genomes and identified classes of SMGCs (lipopeptides and polyketides) that are only present in specific *Bacillus* species^22^. As an example, mining the genomes of the rhizosphere-dwelling *B. amyloliquefaciens* has revealed that it produces a number of antimicrobial and antitumor agents^39–46^. Additionally, the analysis of the phenotypic and genomic properties of *B. thuringiensis* resulted in the identification of *cry* genes and other gene clusters encoding for proteins with toxic phenotypes. This discovery promoted the use of *B. thuringiensis* for insecticidal applications^23,27,47–53^ which are now representing more than 70% of all *Bacillus*-based commercially used biocontrol agents^54^.

The genus also harbors a number of strains with extensively characterized metabolism, most notably the model organism *B. subtilis* 168. This detailed knowledge enabled the use of metabolic and genetic modification methods to transform these strains into efficient microbial cell factories (MCFs)^55^. Additionally, advancements in next-generation sequencing technologies along with the development of computational genome-annotation tools have facilitated mining whole genomes for functional genes and gene clusters that account for unique metabolic and biosynthetic capabilities^56–61^. These genome-mining approaches have become important tools in the quest for new proteins and metabolites^62,63^, and in the increasingly successful efforts to use microbes as cost-efficient agents for industrial production^64–66^. In addition to genome mining, another way of identifying functional differences between taxonomically close strains is through reconstructed genome-scale metabolic networks. This approach has been facilitated by the establishment of large biochemical pathway databases that provide a comprehensive view of the metabolic functions that differ between organisms^67^. For instance, functional genome-scale metabolic models and flux balance analysis successfully identified metabolic functions related to the virulent phenotype of *Staphylococcus aureus* strains in more than 300 different growth-supporting environments^42^.

Our metagenomic-based research initiative reported a rich repertoire of nonribosomal peptide synthetase (NRPS) and polyketide synthase (PKS) sequences (often associated with the synthesis of antimicrobial compounds) derived from strains belonging to the phylum Firmicutes in Red Sea-associated mangrove samples^68^. The ecological uniqueness of this environment, especially its high salinity and temperature, marks its microbial genomic repertoire as putatively attractive for the discovery of unique metabolic and biosynthetic capabilities. Here, we are ranking ten recently sequenced *Bacillus* strains isolated from microbial mat, mangrove mud or barren soil samples taken from Rabigh Harbor Lagoon on the Red Sea shoreline, for their use as MCF platforms for protein and/or metabolite production. We focus on comparing over- and under- represented metabolic reactions in a dataset containing 32 *Bacillus* genomes (22 reference *Bacillus* genomes and the ten Red Sea strain genomes). We also predict and catalog secondary metabolic gene clusters and analyze their homology and co-localization patterns. Finally, in order to further assess the biotechnological potential of these strains, we compared their capacity for protein secretion and sporulation, ranking them against levels detected in the reference strain *Bacillus subtilis* 168. Our results suggest that specific modules of secondary metabolism have evolved in the Red Sea *Bacillus* due to environmental adaptation, and that several of the isolated strains represent promising platforms for development of MCFs.

## Results and Discussion

### *Bacillus* species present in the Red Sea originate from several colonization events

Sequencing the genomes of the ten Red Sea isolates yielded an average of 128,522 reads with a mean length of 9,527 base pairs (bp) and average coverage of 272 (lowest 112 and highest 344). All assemblies are single circular chromosomes without plasmids (except for *B. foraminis* (Bac44), *V. halodenitrificans* (Bac324), *Virgibacillus sp*. (Bac332) and *B. amyloliquefaciens* (Bac57)), with, on average, genome size of 4,536 Kb, 4,483 predicted open reading frames (ORFs), and 29.6 rRNAs (Table 1). Assembly statistics of the raw reads are presented in Supplementary Table S1.

**Table 1:** Summary of sequencing features of the ten Red Sea genomes.

Strain	Species based on 16S RNA assignment	Source	GenBank accession number	Genome size (Mb)	No. contigs	No. ORFs	No. rRNA genes (5S, 16S, 23S)
Bac48	*B. paralicheniformis*	MN	CP023666	4.46	1	4366	24
Bac84	*B. paralicheniformis*	MM	P023665	4.38	1	4306	24
Bac44	*B. foraminis*	MN	CP033044- CP033045	5.43	2	5639	34
Bac144	*B. marisflavi*	BS	CP033051	4.59	1	4440	39
Bac94	*B. halosaccharovorans*	MM	CP033043	5.23	1	5055	58
Bac111	*B. vallismortis*	MM	CP033052	3.96	1	3899	30
Bac57	*B. amyloliquefaciens*	MN	CP033053- CP033054	4.23	2	4107	27
Bac330	*V. dokdonensis*	MN	CP033048	4.46	1	4221	18
Bac332	*Virgibacillus sp*.	MN	CP033046- CP033047	4.56	2	4492	18
Bac324	*V. halodenitrificans*	MN	CP033049- CP033050	4.06	2	4306	24

**Figure 1:**
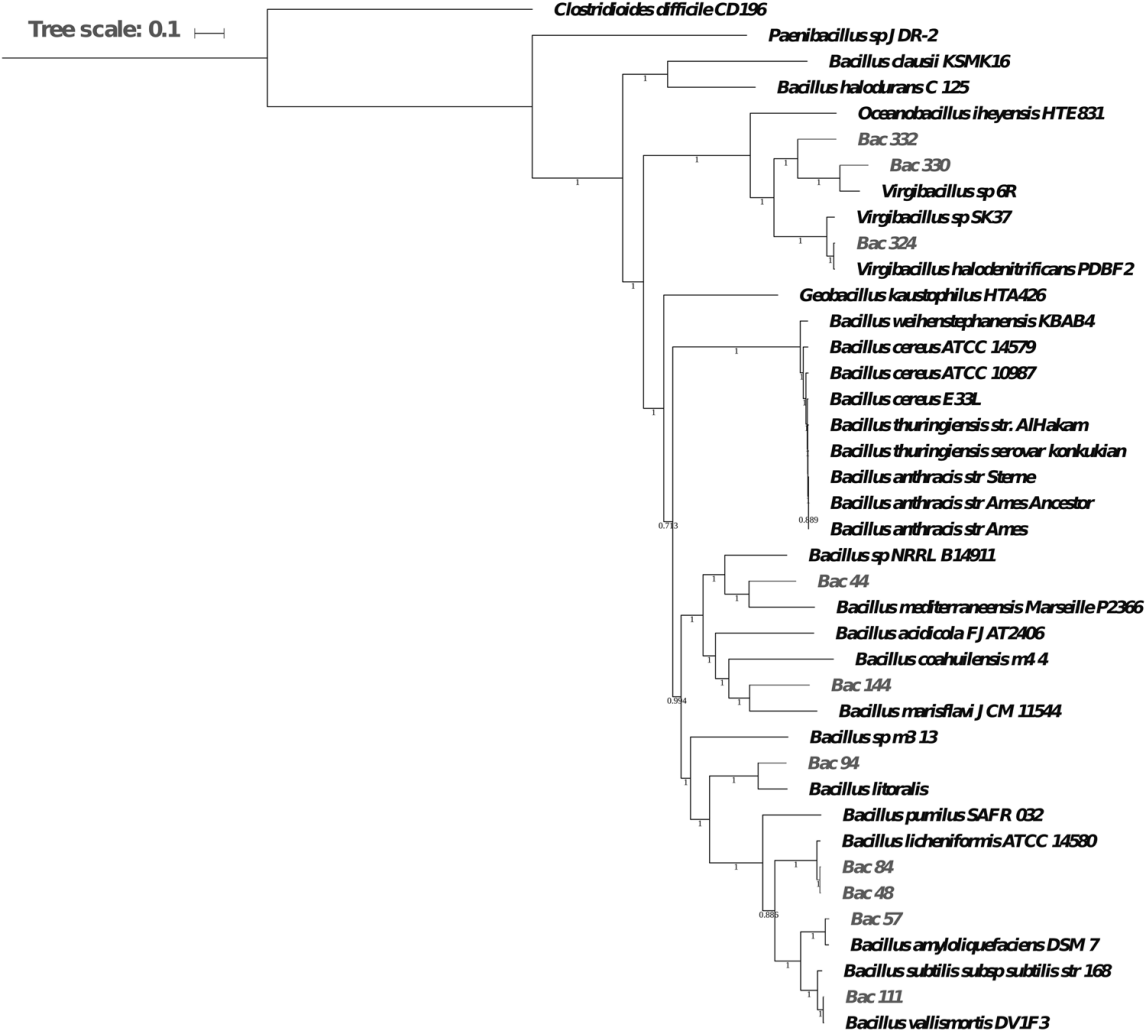
Phylogenetic tree of the ten Red Sea strains and 30 other species. The Red Sea species are displayed in grey while other previously sequenced strains are displayed in black.

To assess the phylogenetic positions of the ten newly sequenced Red Sea species, we ran a whole-genome phylogenetic analysis of those ten species, along with 22 reference *strains* from^69^, and eight *Bacillus* that were found to be closely related to the Red Sea species based on 16S phylogenetic analysis. In total, there are 40 genomes including two outgroups considered in the analysis. The whole-genome phylogeny tree, built using 188 single-copy genes, confirmed that most of the Red Sea species are phylogenetically located within *Bacillus* species (Fig. 1).

Furthermore, since species that underwent single colonization events exhibit consistent patterns in the clades derived from both the phylogenetic tree and their metabolic networks, we further reconciled this phylogenetic tree with the metabolic clades obtained from metabolic reconstructions (see section entitled Convergent evolution of metabolic networks in Bacillus species). This allowed us to hypothesis that since the metabolic clades show different enrichment patterns of distinguishable metabolic pathways, the *Bacillus* species present in the Red Sea have likely gone through several colonization events.

This analysis further showed some species are closely related to other well-studied *Bacillus* species. For example, *B. vallismortis* (Bac111) is closely related to *B. subtilis* 168, whereas *B. paralicheniformis* (Bac84) and *B. paralicheniformis* (Bac48) are closely related to *B. licheniformis* ATCC 14580. Interestingly, *B. subtilis* 168 and *B. licheniformis* ATCC 14580 both have a soil habitat, while the newly sequenced species live in the sea environment. The close relationship between their genomes suggests that some aspects of the metabolism of the newly sequenced species have occurred that enable them to colonize this specific environment. Thus, the *B. paralicheniformis* (Bac84) and *B. paralicheniformis* (Bac48) strains were also used in a separate study focused on the differences in the closely-related *licheniformis* and *paralicheniformis* strains based on predicted biosynthetic capabilities^70^.

### Several Red Sea *Bacillus* species exhibit an above average percentage of their genomes’ genes overlapping with genomic islands

The ten genomes collectively have a total of 2,737,911 bp of DNA sequence overlapping with predicted genomic islands (GIs) regions, with an average length of 273,791 bp per genome (minimum length of 172,668 bp, and a maximum length of 434,257 bp). These GIs are important indicators regarding the discovery of new products as they have been shown to harbor traits that allow strains to adapt to the ecological niche and are enriched with genes associated with SMGCs^71,72^. Therefore, we further calculated and ranked the percentage of the genomes’ genes that are located within predicted GIs for our ten Red Sea strains along with 18 publicly-available *Bacillus* species (Supplementary Table S2). Using the number of DNA sequence base pairs in GIs relative the size of each genome, we found eight of the ten Red Sea strains ranked in the third quartile (5.05–6.69%) and fourth quartile (6.89–19.48%). Also, considering the average of the ‘percentage of the genomes’ genes that are located within predicted GIs’ for all 28 strains is 5.6%, we find five of the Red Sea isolates to have an above average percentage of their genomes overlapping with GIs.

Next, we further investigated what functional properties these GIs provide to the Red Sea strains. The analysis showed that in total 292 genes that are part of GIs overlap with identified biosynthetic genes. Interestingly, four of the Red Sea isolates have horizontally transferred genes falling in NRPS clusters and hybrid PKS/NRPS clusters (Table 2). Specifically, modular genes that are structurally critical for the assembly-line machineries of hybrid PKS/NRPS clusters are part of horizontally transferred genomic regions, indicating the important role these putative GIs play in the synthesis of bioactive compounds. For instance, the NRPS/PKS cluster in *V. dokdonensis* (Bac330) has several structural genes that are predicted to be horizontally transferred, including genes encoding for methyltransferases, peptide synthases, malonyl CoA-acyl carrier protein transacylases and polyketide synthases. All the isolates (i.e., *B. paralicheniformis* (Bac48), *B. amyloliquefaciens* (Bac57), *V. dokdonensis* (Bac330), and *Virgibacillus sp*. (Bac332)) that have modular clusters overlapping with a predicted GI are from mangrove mud samples and not from microbial mat ones. None of the overlapping clusters, except for the cluster for the synthesis of Bacillaene in *B. amyloliquefaciens* (Bac57), has any assigned product using available databases of known SMGCs. Eight of the Red Sea isolates (*B. valismortis* (Bac111), *B. paralicheniformis* (Bac84), *B. amyloliquefaciens* (Bac57), *B. paralicheniformis* (Bac48), *B. marisflavi* (Bac144)*, V. halodenitrificans* (Bac324), *Virgibacillus sp*. (Bac332), *B. foraminis* Bac44) have 13 genes (gene IDs: NP_390764.1, NP_390616.1, NP_388001.1, NP_389359.1, NP_390160.1, NP_389983.1, NP_391547.1, NP_391794.1, NP_388483.2, NP_389974.1, NP_388373.1, NP_388370.1, NP_389162.1) previously reported to be differentially expressed under salt stress conditions during spore outgrowth^73^. This does not mean the other two strains do not have mechanism or SMGCs that allow them to thrive in a salt stressed environment. Specifically, Bac330 is placed into a clade that is statistically enriched with reactions for the biosynthesis of ectoine, a known osmoprotectant. Also, Bac94 has the highest number of unique BGCs, that are not shared with any species, most of which are not assigned to known products and may be contributing to the strains’ salt tolerance. Collectively, these results show that the identified horizontally transferred regions with functionally relevant genes, such as the ones involved in salt tolerance and bioactive compounds, are putative indicators of the environment-specific increased fitness of these Red Sea isolates.

**Table 2:** Predicted genomic islands and prophage regions in the Red Sea genomes showing their overlap with biosynthetic genes

Genome	GI %	Prophage %	Overlap with biosynthetic genes		Assigned product
			Number of genes	Cluster type(s)	
*B. paralicheniformis (*Bac48)	5.05	2.43	38	terpene, trans-acyltransferase PKS/NRPS	—
*B. paralicheniformis* (Bac84)	5.77	3.20	21	terpene, bacteriocin	—
*B. foraminis* (Bac44)	2.89	2.75	0		
*B. marisflavi* (Bac144)	5.29	0.61	53	terpene	—
*B. halosaccharovorans (*Bac94)	4.31	0.28	19	terpene	—
*B. vallismortis* (Bac111)	6.19	0.88			—
*B. amyloliquefaciens* (Bac57)	9.91	7.04	46	phosphonate, trans-acyltransferase PKS/NRPS, lantipeptide, NRPS	Bacillaene
*V. dokdonensis* (Bac330)	6.95	3.11	43	NRPS, NRPS-PKS	—
*Virgibacillus sp*. (Bac332)	9.39	4.11	72	trans-acyltransferase PKS/NRPS, NRPS	
*V. halodenitrificans* (Bac324)	5.19	3.68	0		—

### Several Red Sea *Bacillus* strains exhibit potential as protein-secreting cell factories

**Figure 2:**
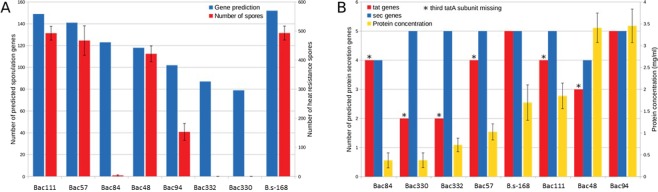
Evaluation of sporulation (**A**) and protein secretion (**B**) in the Red Sea strains through gene prediction and *in vitro* measurements. Strains with the third TatA subunit missing, are indicated with *.

*Bacillus* species are extensively used for production of industrial enzymes. In order to qualify for such applications, a candidate strain would ideally be non-sporulating and have efficient protein secretion machinery. We provide a preliminary bioinformatics analysis of the potential sporulation of the species as sporulation is a complex developmental process, involving several hundred genes^69,74^. The caveat here is that the presence of a high number of sporulation genes merely indicates that the species has sporulation potential, it does not necessarily correlate to the number of spores, as expression of these genes are not taken into account. Thus, we further experimentally evaluated the Red Sea species along these lines, by comparing them to the standard laboratory strain *B. subtilis* 168. Three of the species (*B. foraminis* Bac44, *B. marisflavi* (Bac144), and *V. halodenitrificans* (Bac324)) did not grow on standard laboratory media, thus Fig. 2A (and Fig. 2B) show only results for seven Red Sea strains.

Figure 2A show no correlation between the number of genes involved in sporulation and the number of spores produced by the different species. However, it does show the higher the number of sporulation genes present in a species, the higher the chance that key sporulation genes are not missing, as is evident in the *Virgibacillus* strains, and sporulation potential is more likely. That is, *Virgibacillus* strains Bac332 and Bac330 exhibited the least number of sporulation genes, and were completely incapable of sporulating, under the conditions tested. This could be related to the fact that in both species, despite the presence of many sporulation genes, the gene *spo0B* is missing. This gene is a key element in the phosphorelay regulating sporulation initiation^75^. Its absence has already been reported to arrest sporulation in phase 0^75^. No clear explanation has been found concerning the extremely low level of sporulation of *B. paralicheniformis* (Bac84) under the conditions tested. Further studies will be needed to explain this phenomenon. Globally, it is interesting that none of the Red Sea species sporulate more than *B. subtilis 168* under the conditions used.

Protein secretion in *Bacillus* species is mainly controlled by two systems: the twin arginine translocation (Tat) and general secretion (Sec) systems, which transport folded and unfolded proteins, respectively, across the membrane. In *B. subtilis*, both systems involve only a small number of genes: *tatAd*, *tatAy, tatAc*, tatCd and tatCy; and *secA*, *secY*, *secE*, *secG* and *secDF*. Comparing the genes present in the Red Sea species and the actual protein secretion (Fig. 2B), no clear correlation has been found between the presence of genes from the Tat and Sec systems, and the actual protein secretion capabilities. It is nevertheless worth noting that two of the species from the Red Sea strains secrete twice as much protein as *B. subtilis 168* when they are grown in the LB medium. One of these species, *B. halosaccharovorans (*Bac94) possesses complete Tat and Sec systems. These results serve as positive features for the selection of *B. halosaccharovorans* (Bac94) and *B. paralicheniformis* (Bac48) as MCFs optimized for enzyme production, as well as for further study protein of secretion pathways.

### Convergent evolution of metabolic networks in *Bacillus* species

To better characterize the metabolism of the 32-studied species, and to discover eventual metabolic features specific to the ten Red Sea species, we reconstructed all their metabolic networks. These metabolic networks contained from 671 to 1,398 reactions, with an average of 1,238 reactions (median: 1,291 reactions) and contained from 1,050 to 1,897 different metabolites involved in these reactions, with an average of 1,667 metabolites (median: 1,715 metabolites). It is interesting to note that the three networks containing the most metabolic reactions correspond to the Red Sea species. Globally, the Red Sea species possess on average 57 more reactions than the other species (1,277 vs. 1,220 reactions) and on average 75 more metabolites (1,719 vs. 1,644 metabolites). The reactions are inferred based on metabolic genes. We used between 477 and 1,042 genes per species, 903 on average (median: 941).

These numbers could be compared to the most complete existing metabolic model of *B. subtilis* 168, *i*Bsu1103^76^. This metabolic model contains 1,437 reactions associated with 1,103 genes. For comparison, our reconstruction of the metabolic network of *B. subtilis* 168 contains 1,366 reactions associated with 1,006 genes. The numbers are similar and the small difference could be attributed to the absence of gap-filling and the fact that, in our case, the manual curation effort was less intense compared to the one performed for *i*Bsu1103.

**Figure 3:**
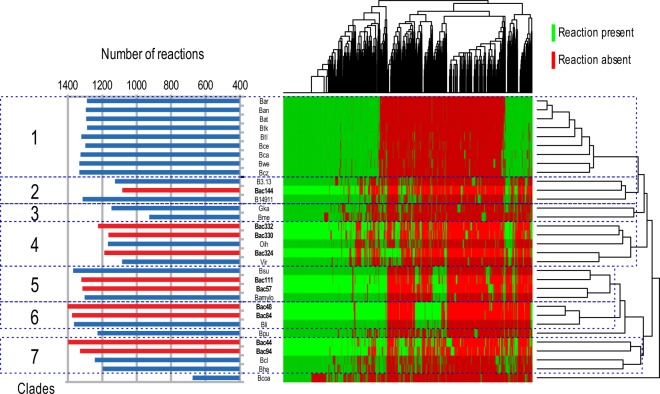
Metabolic networks reconstruction. The left part of the figure corresponds to the number of reactions present in the metabolic networks. The right part of the figure corresponds to a clustering performed on the 32 species based on the presence and absence of reactions. The 32 species have been divided into seven metabolic clades based on this clustering.

To find signatures of metabolism in *Bacillus* species, we compared the reactions present in the different metabolic networks. Clustering the genomes, represented in Fig. 3, has been made based on the presence/absence of reactions. Based on this clustering, several “metabolic clades” appeared clearly. We decided to divide the 32 species into seven different metabolic clades based on the species dendrogram obtained. Based solely on the dendrogram, the clades 5 and 6 may have been merged, but we decided to split them to understand the meaning of the group of reactions predominantly present in the clade 6. *B. coahuilensis* m4–4 was not included in the analysis because of the low quality of its genome, and hence the expected low quality of its metabolic network. *B. pumilus* SAFR-032 was also not included in this analysis since it appeared to be too distant to all the other clades and the subsequent statistical analysis would not be relevant.

We first compared the obtained dendrogram and the previous phylogenetic analysis. Some of the metabolic clades are conserved compared to the phylogenetic clustering (clades 1, 4, 5 and 6). On the other hand, metabolic clades 2, 3 and 7 do not correspond to a phylogenetic clustering. This could be the sign of a convergent evolution of the metabolism of these species, which could have evolved to adapt to special environmental conditions. To facilitate the composition of the metabolic networks, a binomial test was performed on each reaction to find if a given reaction is statistically over-represented or under-represented in a given clade (Supplementary Dataset S1). The obtained reactions have been grouped in pathways as defined in the MetaCyc database.

From this analysis, several hypotheses can be drawn about the metabolism of the analyzed species. For example, the metabolic clade 4 possesses statistically more reactions involved into ectoine biosynthesis. Ectoine is already known for their osmoprotectant power^77,78^, suggesting that *Virgibacillus* strains Bac332, Bac330, Bac324, *sp. SK37* and *Oceanobacillus iheyensis HTE831* are highly tolerant against osmotic stress. The same species have more reactions involved in glucuronoarabinoxylan degradation compared to others, which can be linked to utilization of plant cell wall polysaccharides. Metabolic clades 5 and 6 seems also prone to degradation of plant cell walls with an over-representation of reactions involved into rhamnogalacturonan type I degradation and D-galacturonate degradation. This assumption is validated with the presence of *B. licheniformis*, *B. subtilis* and *B. amyloliquefaciens* in those clades, that are all known to be associated with plant and plant material in nature^39,79^. These results are also consistent with a previous study^69^ where the evolutionary and functional relationships between twenty complete and draft *Bacillus* genomes are compared and found that most of the metabolic variation between the strains was stemming from genes related to functions necessary for adapting to the environments from which they were isolated.

In metabolic clades 2, 3, and 7, the most represented metabolic pathways were carbon metabolism, amino acid metabolism, and nucleotide metabolism. In clade 2 we also find the dimethylsulfide (DMS) degradation pathway that is a significant contributor of sulfur. Degradation of dimethylsulfoniopropanoate (DMSP) osmolytes in marine, estuarine, and salt marsh systems are the primary source of DMS. DMSP was shown to be degraded by several bacterial types from salt marsh sediments^80,81^. Also, metabolic clades 2 and 7 possess a few antibiotic biosynthesis pathways e.g., Kanamycin biosynthesis in clade 2, and Paromomycin and Mithramycin biosynthesis in clade 7^82^. There are also some interesting reactions involved in detoxification, such as furfural degradation in clade 2^83^, and a reaction related to mercury detoxification pathways in clade 3^84^. These pathways may be providing a selective advantage for these Bacillus species to thrive in the Red Sea environment.

Moreover, we also investigated if any reactions that are commonly shared by the 10 Bacillus species are associated with adaptation to the local environment. We found no reactions shared by all ten Red Sea *Bacillus* species while being absent from other *Bacillus* species. Figure 3 depicts this result as well, where the 10 *Bacillus* species do not cluster together based on their reactions. If we choose a looser threshold to select reactions that are enriched in at least 75% of the networks of Red Sea species and are absent in at least 90% of the other networks, we filter down the number of reactions to 17 (Supplementary Table S3). The low number of reactions hinders the inference of possible functional pathways these reactions might be part of. Manual inspection of the functions of these reactions revealed that one of these reactions gives relevance to pectate lyase, which might be related to the prevalence of mangrove trees in the proximate vicinity of the isolation site. However, we would like to note that the exclusive presence of these reactions in most of the Red species, is not necessarily a discriminative feature of the uniqueness of these species. It is indeed the collective analysis of the distribution of reactions and corresponding pathways, such as we did in Fig. 3, that succeeds in drawing the lines between all the analyzed *Bacillus* species in general, including the Red Sea *Bacillus* isolates.

### The potential secondary metabolism of the Red Sea strains suggests the production of novel active molecules

**Figure 4:**
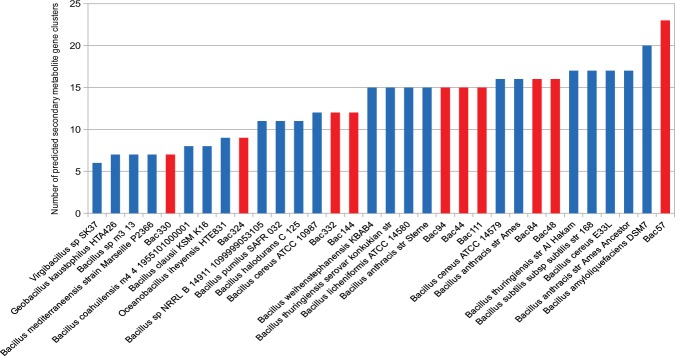
Number of predicted secondary metabolic gene clusters in the genomes of the Red Sea strains (red) and 22 other Bacillus genomes (blue).

We noted a big diversity in the number of SMGCs predicted in different *Bacillus* species, ranging from 6 to 23 gene clusters per species (Fig. 4). On average there are 13 gene clusters per species (the median is 15). For example, *Virgibacillus* strains Bac330 and Bac332 are very close from a phylogenetic and metabolic point of view, but the first has almost twice the number of gene clusters compared to the second. The species containing the most SMGCs is the Red Sea strain *B. amyloliquefaciens* (Bac57) which has ten more gene clusters than the average.

To compare more precisely the secondary metabolism of different species, we looked for gene clusters that are shared between several species. An annotation of known gene clusters has also been performed. In total, 417 gene clusters are present in the 32-studied species. Since the same gene cluster can be present in different species, we can distinguish in total 200 different gene clusters: 55 are shared between at least two species, while 145 are unique for a given species. 21 of those 200 gene clusters are known, while 179 are still unknown, highlighting a huge potential of new discoveries in future. In particular, 54 unknown gene clusters are only present in the genomes of the Red Sea species (Supplementary Fig. S1).

**Figure 5:**
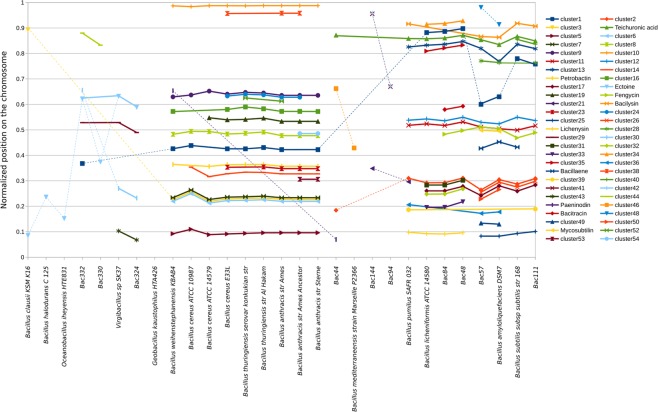
Co-localization of gene clusters using the normalized position for each SMGC based on the length of the chromosome and the position of the middle of the SMGC. Plain lines join co-localized SMGCs, dotted lines join SMGCs that are not co-localized.

Since complete genomes are available for most of the studied species (29 out of 32), we next studied the localization of gene clusters in different species. Studying the localization of the SMGCs in the genomes could serve as a proxy to study the evolution of these gene clusters in the *Bacillus* species. Specifically, the colocalization of the same SMGCs on random genomic positions, as these SMGCs are reported to be putative indicators of horizontal gene transfer or chromosomal rearrangement in close species^85,86^. We found that most of the gene clusters shared by at least 2 species co-localize in their genomes, implying that these gene clusters appeared in one of their common ancestors (Fig. 5). Among the 54 clusters of gene clusters shared by at least two species having their complete genomes, 38 are strictly co-localized, four have genes that co-localize in at least two species but are also placed somewhere else in other genomes, and 12 are randomly appearing in all the genomes. Interestingly, while the SMGCs present in clusters No. 49 and 51 perfectly co-localize in the genomes of *B. amyloliquefaciens DSM7* and *B. amyloliquefaciens* (Bac57), the SMGCs present in clusters No. 48 and No. 50, also shared by both species, are not co-localized in the genome with respect to their normalized positions on the chromosome. Cluster No. 37, assigned to the production of the antibiotic Bacillaene is present and co-localizes in the genomes of *B. amyloliquefaciens* (Bac57), *B. amyloliquefaciens DSM7* and *B. subtilis subsp subtilis str.168*, while it is not present in the genome of *B. vallismortis* (Bac111) suggesting a loss of this cluster in that species during evolution. Conversely, the SMGC No. 21 is present in *B. marisflavi* (Bac144) and *B. pumilus SAFR 32* and not co-localized, suggesting an independent apparition of this gene cluster in these species. In the same manner, cluster No. 3 is present at the same position in genomes from the *B. cereus* group suggesting an apparition in the common ancestor of these species, while this cluster is also present in *B. clausii* KSM K16 at another position suggesting an independent apparition in this species. The exact same mechanism seems to exist in the *B. subtilis* group and *B. foraminis* (Bac44), with the SMGC present in the cluster No. 2. The raw results of this analysis are presented in Supplementary Table S4.

Concerning the Red Sea species, they possess in total 95 different SMGCs. Among them, 38 are shared by at least one other species, while 57 are unique. We have been able to assign a product to ten of the shared gene cluster (29% of them) and five of the unique ones (9%). It is worth noting that only three SMGCs present in more than one species are shared solely among Red Sea species. Among the 15 SMGCs from the Red Sea species, we can see an enrichment of SMGCs involved in the production of antibiotics and lantibiotics, representing 66% of the identified SMGCs. The three identified SMGCs shared by the biggest number of species, including Red Sea Species, concern protections against environmental changes. For example, we can identify the genes involved in production of Bacillibactin, a siderophore^87^, teichuronic acid, a part of the cell wall produced in low-phosphate conditions^88^, or ectoine, which serves as protection against osmotic stress.

Among the identified SMGCs, those that are less spread among the studied species mostly correspond to antibiotics, lantibiotics and antifungals. We can, for example, cite the identification of gene clusters involved in production of an antibiotic bacitracin, a class II lantibiotic pseudomycoicidin, or an antifungal fengycin^89^ shared by four out of the ten Red Sea species.

This highlights once again a potential of new secondary metabolites discoveries, especially among molecules that would be produced by gene clusters uniquely present in these species. For example, it is worth noting that the species *B. foraminis* (Bac44), *B. halosaccharovorans (*Bac94) and *B. marisflavi* (Bac144), while possessing between 12 and 15 SMGCs, share only two of these gene clusters with other species. Experimental metabolite profiling is needed to address these hypotheses and truly check the different species capacity for secondary metabolite synthesis.

## Concluding Remarks

Identifying environment-specific phenotypes in newly isolated species is one way to hunt for potentially efficient MCFs. Here, through comparative analysis, we first established the existence of notable indicative patterns of convergent evolution in the genomes of 10 sequenced Red Sea strains and other representative *Bacillus* species. Further experimental evaluation of key properties for optimal microbial platforms revealed that some of the Red Sea strains are efficient protein secretors surpassing the model strain *B. subtilis* 168. From a bioinformatics perspective, we also evaluated if the presence of a complete set of genes represents a more positively-correlated phenotype compared to those without, given that the presence of these genes is requirements for their potential activation and expression within organisms. The bioinformatic analysis confirmed the genomic enrichment of the genomes with secretion system-related and sporulation-related genes, do not necessarily add more evidence to the organism’s potential protein secretion capacity. By implementing a ranking system that leverages the available completely sequenced genomes, we evaluated the production potential of the isolated strains, taking into account critical driving features, such as their overall metabolic similarity, the distribution of biosynthetic genes and the prevalence of horizontally transferred genes of biosynthetic relevance. The comparative analysis primarily pointed towards *B. paralicheniformis* (Bac48) and *B. halosaccharovorans* (Bac94) as the top candidates for further probing and optimization for the development of efficient MCFs.

## Material and Methods

### Sampling, isolation and purification of bacterial strains

The sampling, isolation and purification of Red Sea mangrove strains were previously described in^90^. Strains were isolated from samples collected from the Rabigh Harbor Lagoon by the Red Sea in Saudi Arabia (39°0′35.762′′E, 22°45′5.582′′ N). Bac44, Bac48, Bac57, Bac324, Bac330 and Bac332 were isolated from samples taken from mangrove mud, while the other samples were isolated from microbial mat (Bac84, Bac94, and Bac111) and barren soil (Bac144) in close proximity.

### DNA extraction, sequencing, assembly and annotation

Sigma Gen Elute Bacterial Genomic DNA Kit (Sigma, USA) was used to extract Genomic DNA following the manufacturer’s protocol and the PowerClean Pro Clean-Up Kit (MO BIO, USA) was utilized as another purification step. DNA purification was assessed by overnight gel electrophoresis and NanoDrop (Thermo Fisher Scientific, USA). DNA was quantified using Qubit 2.0 (Life Technologies, Germany). The whole genome sequencing of the ten Red Sea strains was done on the PacBio RS II sequencing platform (Pacific Biosciences, USA) in the Core Laboratory sequencing facility at King Abdullah University of Science and Technology (KAUST). P6-C4 chemistry was used to sequence the large-insert libraries in single-molecule real-time (SMRT) sequencing cells. The assembly of the genomes was carried out using PacBio’s SMRT Analysis pipeline v2.3.0 setting the GenomeSize parameter to 6 Mb. All other parameters were set to default. All assembled genomes were examined for indications of circularization using Gepard^91^, then circularized, if applicable, using minimus2^92^. To polish the assembly, SMRT Analysis Resequencing protocol was used in multiple rounds until convergence. The Automatic Annotation of Microbial Genomes pipeline (AAMG)^93^ was used to annotate the genomes. Finally, genomic islands (GIs) and prophages were predicted using IslandViewer v4^94^ and PHASTER^95^, respectively.

### Phylogenetic placement and clustering

The phylogeny analysis was performed based on single-copy genes shared amongst 40 representative strains including the 10 Red Sea ones. The single-copy genes were aligned using MUSCLE v3.8.31^96^ and the alignments were concatenated using FASconCAT-G v1.02^97^. ProtTest3 v.3.4.2^98^ was then used to predict the amino-acid replacement model. Finally a maximum likelihood tree was built using PhyML v3.1.^99^ under the LG + I + G (LG) model as recommended by ProtTest3, with 100 bootstrap replicates.

### Identification of protein secretion and sporulation genes

To identify protein secretion and sporulation genes, homologous genes to those present in *B. subtilis* 168 were identified using BLASTp and the bidirectional-best blast hits (BBH) method. Genes related to sporulation and protein secretion functions were retrieved from SubtiList (http://genolist.pasteur.fr/SubtiList/)^100,101^. A gene was considered to be present in the genome if it has a bidirectional hit with its homologue in *B. subtilis*. The minimum e-value was set to be 10^−5^. All top hits had an e-value of 10^−20^ or less. If a gene is absent in a genome, it was given a coverage value of 0.

### Metabolic network reconstruction

The metabolic networks were reconstructed using the MetaCyc^102^ database. For every reaction in MetaCyc, the sequences of the enzymes catalyzing the reactions were retrieved. In cases where, only one enzyme sequence was available for a given reaction, a blast alignment^103^ was run between this sequence and the predicted proteome of a given species (bit-score threshold of 100). If, however, multiple enzyme sequences were available for a given reaction, an HMM model characterizing those sequences was reconstructed using HMMer^104^. This model was then searched against the predicted proteome using the same bit-score threshold of 100.

Metabolic clades were identified based on a manual inspection of the dendrogram associated with the heatmap describing the presence/absence of reactions. To find over- and under-represented reactions per metabolic clade in the heat map, a binomial test was run for each reaction, comparing the number of positive occurrences between the considered clade and the other species. A threshold of 10^−5^ was chosen to consider a reaction as over- of under-represented in a given clade.

### Secondary metabolic gene clusters prediction and analysis

Predictions of secondary metabolic gene clusters was performed on the public web version of antiSMASH 4.0 (http://antismash.secondarymetabolites.org)^105^. The ClusterFinder algorithm was used to predict the borders of biosynthetic gene clusters. A manual curation was performed to define more precisely the boundaries of the gene clusters based on similarities with known gene clusters present in the MIBiG database. Some of the predicted gene clusters have been split in two different gene clusters when sufficient information was available to make such a decision. The secondary metabolite product of a given gene cluster was considered as identified if this gene cluster showed at least 60% of similarities with one characterized gene cluster present in the MIBiG database. MultiGeneBlast^106^ was used to infer similarities between all predicted gene clusters in the Bacillus species. Two gene clusters were considered as similar if they show at least 60% of similarities with each other. To identify co-localization of gene clusters, a normalized position was computed for each SMGC based on the length of the chromosome and the position of the middle of the SMGC. This was only computed for the species possessing a complete chromosomal sequence.

### Bacterial strains and media

All strains were cultured in Luria-bertani (LB) broth at 37 °C in shaking flask. Minimal media M9^107^ was used for secretion essays and the Difco Sporulation Medium (DSM)^108^ for sporulation essays. When relevant, kanamycin (25 µg/ml) and erythromycin (1 µg/ml) were added for the *Bacillus* strains genetic transformation.

### Sporulation assay

The overnight LB cultures of the *Bacillus* strains at 37 °C were diluted to OD600 0.1 in preheated liquid DSM medium^108^ and cultured under shaking conditions (200 rpm) at 37 °C to OD600 of two (around 24 hours of culture). 500 µl of the culture was heated at 80 °C for 10 minutes. Serial dilutions from the heated samples were made in 0.9% NaCl (from 10–1 to10–7) and 100 µl of the material from dilution with 10–3 to10–7, plated in LB and incubated for 36 h at 37 °C. After incubation the colonies were counted on plates and used as the number corresponding to heat-resistant spores. Three biological replicates were performed, providing similar results. One representative biological replicate for each *Bacillus* strain is reported with standard deviation of variables from three technical replicates.

### Protein secretion assay

The tested *Bacillus* strains were grown overnight in culture using LB at 37 °C and 200 rpm shaking speed. 20 ml of minimal media (M9), which was supplemented with 0.4% glucose^107^, were inoculated to OD600 of 0.1. At the late exponential phase of growth, 3 Mm of the protease inhibitor Phenylmethylsulfonyl fluoride (PMSF) was added to the cultures to prevent proteolytic digestion and then the cell was grown for 24 h to OD600 of 2. The cultures were centrifuged at (8000 × g) for ten minutes and the supernatants were filtered with 0.2 nm nitrocellulose to eliminate the rest of cells. To precipitate the secreted proteins, 50% (w/v) of ammonium sulphate was added to the supernatants and kept on ice for 1 hour. The mixtures were then centrifuged at 25000 × g for 20 minutes. The precipitated proteins were resuspended in PBS buffers at pH 6.8. The proteins concertation was measured using NanoDrop, and compared with samples of M9 media without bacteria treated with the same procedure for culture and protein precipitation. One representative biological replicate is reported from three technical replicates.

## Supplementary information

Supplementary Figures and Tables

Supplementary Dataset S1

## Data Availability

All data used in this study have been included in this article and its additional files.
