# Supplementary material for: Comparative genomics study reveals Red Sea Bacillus with characteristics associated with potential microbial cell factories (MCFs)
Source: Sci Rep. 2019 Dec 17;9:19254. doi: 10.1038/s41598-019-55726-2 (PMC6917714; doi:10.1038/s41598-019-55726-2)
Supplement: Supplementary file 1 — Supplementary Figures and Tables [file 41598_2019_55726_MOESM1_ESM.docx]

**SUPPLEMENTARY INFORMATION**

**Comparative genomics study reveals Red Sea *Bacillus* with characteristics associated with potential microbial cell factories (MCFs)**

Othoum G ^1*^, Prigent S ^2*^, Derouiche A ^2^, Shi L ^2^, Bokhari A ^3^, Alamoudi S ^4^, Bougouffa S ^1^, Gao X ^1^, Hoehndorf R ^1^, Arold ST ^1^, Gojobori T ^1,3^, Hirt H ^3^, Lafi FF ^5,1^, Nielsen J ^2,6,7^, Bajic VB ^1^, Mijakovic I ^2,6#^, Essack M ^1#^

^1^ Computational Bioscience Research Center (CBRC), King Abdullah University of Science and Technology (KAUST), Thuwal 23955-6900, Kingdom of Saudi Arabia.

^2^ Department of Biology and Biological Engineering, Division of Systems & Synthetic Biology, Chalmers University of Technology, Kemivägen 10, 41296 Gothenburg, Sweden

^3^ Biological and Environmental Sciences and Engineering Division (BESE), King Abdullah University of Science and Technology (KAUST), Thuwal 23955-6900, Kingdom of Saudi Arabia.

^4^ Department of Biology, Science and Arts College, King Abdulaziz University, Rabigh, 21589 Kingdom of Saudi Arabia

^5^ College of Natural and Health Sciences, Zayed University, 144534, Abu-Dhabi, United Arab Emirates.

^6^ Novo Nordisk Foundation Center for Biosustainability, Technical University of Denmark, 2800 Lyngby, Denmark.

^7^ Science for Life Laboratory, Royal Institute of Technology, Solna, Sweden

^#^ To whom correspondence should be addressed. Tel: +966 (54) 470 0746; Email: [magbubah.essack@kaust.edu.sa](mailto:magbubah.essack@kaust.edu.sa); Email: [ivan.mijakovic@chalmers.se](mailto:ivan.mijakovic@chalmers.se)

^*^The authors wish it to be known that, in their opinion, the first two authors should be regarded as joint First Authors

ME : [magbubah.essack@kaust.edu.sa](mailto:magbubah.essack@kaust.edu.sa)

GO : [ghofran.othoum@kaust.edu.sa](mailto:ghofran.othoum@kaust.edu.sa)

SP : [prigent@chalmers.se](mailto:prigent@chalmers.se)

AD : [abdder@chalmers.se](mailto:abdder@chalmers.se)

LS : [slei@chalmers.se](mailto:slei@chalmers.se)

AB : [ameerah.bokhari@kaust.edu.sa](mailto:ameerah.bokhari@kaust.edu.sa)

SA : [saalamudi@kau.edu.sa](mailto:saalamudi@kau.edu.sa)

SB : [salim.bougouffa@kaust.edu.sa](mailto:salim.bougouffa@kaust.edu.sa)

XG : [xin.gao@kaust.edu.sa](mailto:xin.gao@kaust.edu.sa)

RH : [Robert.Hoehndorf@kaust.edu.sa](mailto:Robert.Hoehndorf@kaust.edu.sa)

STA : [stefan.arold@kaust.edu.sa](mailto:stefan.arold@kaust.edu.sa)

TG : [takashi.gojobori@kaust.edu.sa](mailto:takashi.gojobori@kaust.edu.sa)

HH : [heribert.hirt@kaust.edu.sa](mailto:heribert.hirt@kaust.edu.sa)

FFL : [Feras.Lafi@zu.ac.ae](mailto:Feras.Lafi@zu.ac.ae)

JN : [nielsenj@chalmers.se](mailto:nielsenj@chalmers.se)

VBB : [vladimir.bajic@kaust.edu.sa](mailto:vladimir.bajic@kaust.edu.sa)

IM : [ivan.mijakovic@chalmers.se](mailto:ivan.mijakovic@chalmers.se)

# Supplementary Figures
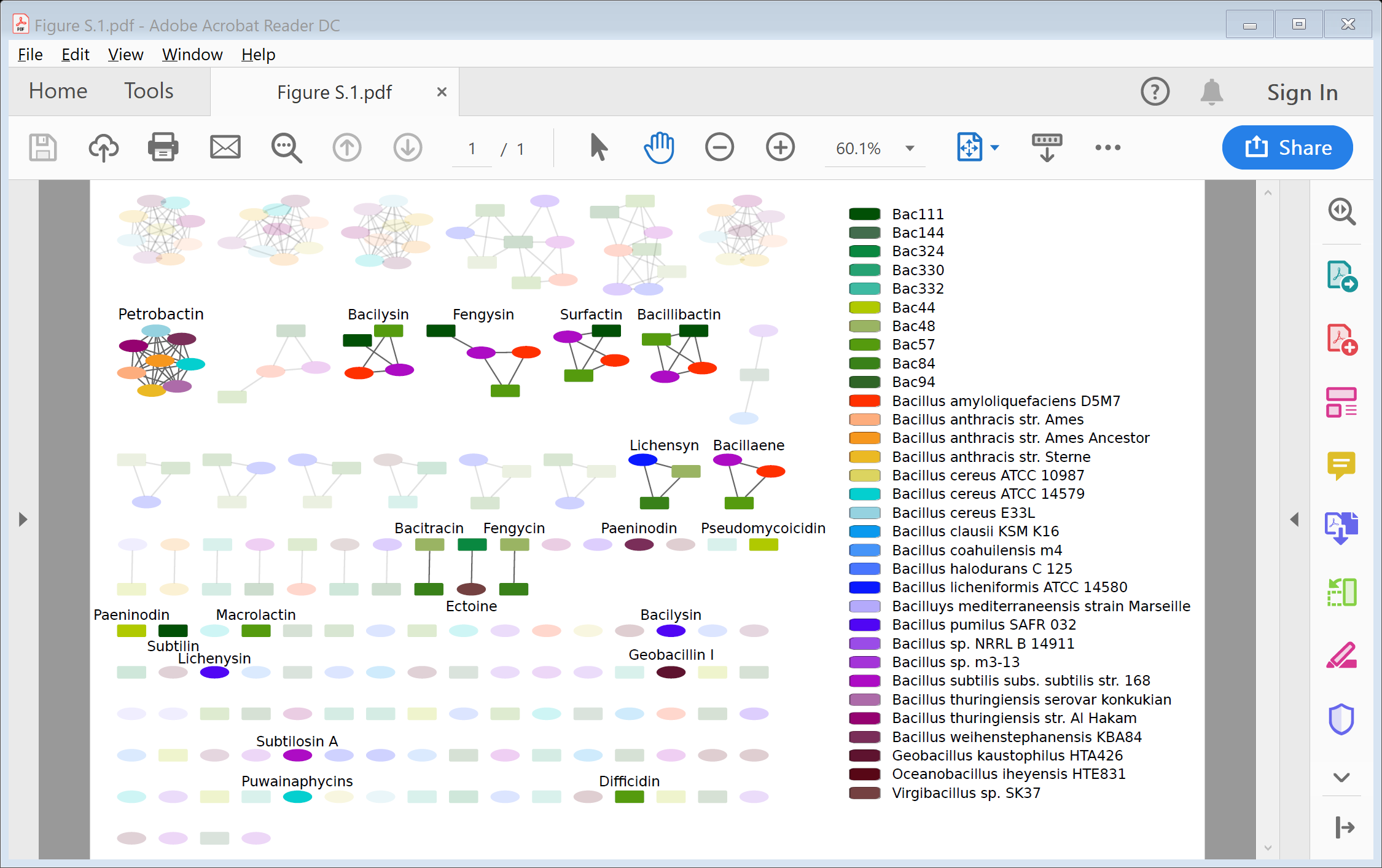


**Supplementary Figure S1**. Co-occurrence network of secondary metabolism gene

clusters. Unknown gene clusters are marked by a transparent color. Red Sea species are

displayed in boxes in different shades of green. Clusters from other species are displayed as

ellipses. The product of a given secondary-metabolic gene cluster is displayed when

it has been identified.

# Supplementary Tables

**Supplementary Table S1**. Assembly statistics of raw sequenced reads.

* plasmid size: 551,486, ** contig 1 size: 8,648, *** contig 2 size: 65,691, **** contig 3 size: 312,876.

|  | **Polymerase Reads** | | |  | |  | |  | | **Subreads** |  | | |  | |  |  | | |  |
| --- | --- | --- | --- | --- | --- | --- | --- | --- | --- | --- | --- | --- | --- | --- | --- | --- | --- | --- | --- | --- |
| **Species** | **Bases (bp)** | | | **Reads** | | **N50 (bp)** | | **Average Length (bp)** | | **Subreads** | **Bases** | | | **N50 (bp)** | | **Average Length (bp)** | **Genome Size** | | | **Coverage** |
| Bac48 | 1,334,497,375 | | | 86,252 | | 22,785 | | 15,472 | | 138,867 | 1,331,266,339 | | | 12,578 | | 9,586 | 4464381 | | | 298 |
| Bac84 | 1,197,038,773 | | | 75,932 | | 21,104 | | 15,764 | | 108,978 | 1,194,885,446 | | | 15,215 | | 10,964 | 4376831 | | | 273 |
| Bac44* | 1,396,854,389 | | | 88,848 | | 22,380 | | 15,721 | | 146,170 | 1,393,125,258 | | | 12,595 | | 9,530 | 5426180 | | | 257 |
| Bac144 | 518,373,336 | | | 75,822 | | 9,008 | | 6,836 | | 81,831 | 517,423,429 | | | 8,436 | | 6,323 | 4591517 | | | 113 |
| Bac94 | 1,322,684,968 | | | 85,294 | | 21,346 | | 15,507 | | 127,321 | 1,320,002,384 | | | 14,117 | | 10,367 | 5230624 | | | 252 |
| Bac111 | 1,336,239,096 | | | 82,020 | | 22,177 | | 16,291 | | 117,076 | 1,333,980,830 | | | 15,887 | | 11,394 | 3958372 | | | 337 |
| Bac57** | 1,459,810,320 | | | 86,164 | | 26,095 | | 16,942 | | 163,725 | 1,455,135,660 | | | 11,874 | | 8,887 | 4226250 | | | 344 |
| Bac330 | 1,130,712,574 | | | 78,852 | | 20,738 | | 14,339 | | 141,676 | 1,126,723,067 | | | 10,257 | | 7,952 | 4456326 | | | 253 |
| Bac332*** | 1,327,058,771 | | | 81,344 | | 24,075 | | 16,314 | | 148,002 | 1322993204 | | | 11,784 | | 8,939 | 4561556 | | | 290 |
| Bac324**** | 1,265,911,284 | | | 73,935 | | 22,789 | | 17,121 | | 111,571 | 1,263,526,786 | | | 15,664 | | 11,324 | 4063118 | | | 311 |
|  | |  |  | |  | |  | |  | | |  |  | |  | | |  |  |  |
|  | |  |  | |  | |  | |  | | |  |  | |  | | |  |  |  |
|  | |  |  | |  | |  | |  | | |  |  | |  | | |  |  |  |
|  | |  |  | |  | |  | |  | | |  |  | |  | | |  |  |  |

**Supplementary Table S2.** Percentage of strains’ genomes and genes that are located within predicted GIs and prophages for ten Red Sea strains along with 18 publicly-available Bacillus species

| **Genome** | **GI region(s) size** | **Prophage(s) region size** | **GI%** | **Prophage(%)** |
| --- | --- | --- | --- | --- |
| Bacillus anthracis str. Ames chromosome | 61,113 | 207,700 | 1.17 | 3.97 |
| Bacillus thuringiensis str. Al Hakam | 72,454 | 30,200 | 1.38 | 0.57 |
| Bacillus anthracis str. Sterne chromosome | 86,465 | 190,200 | 1.65 | 3.64 |
| Bacillus cereus E33L | 107,434 | 88,400 | 2.03 | 1.67 |
| Bacillus pumilus SAFR-032 | 86,757 | 32,700 | 2.34 | 0.88 |
| Oceanobacillus iheyensis HTE831 | 99,416 | 15,900 | 2.74 | 0.44 |
| Bac44 | 172,668 | 26,800 | 2.89 | 2.75 |
| Bacillus thuringiensis] serovar konkukian str. 97-27 | 166,585 | 35,100 | 3.18 | 0.67 |
| Bacillus anthracis str. 'Ames Ancestor' | 173,195 | 164,600 | 3.31 | 3.15 |
| Bacillus cereus ATCC 14579 | 192,120 | 140,400 | 3.55 | 2.59 |
| Bacillus weihenstephanensis KBAB4 | 205,490 | 127,400 | 3.90 | 2.42 |
| Bac94 | 225,344 | 14,400 | 4.31 | 0.28 |
| Bacillus cereus ATCC 10987 | 244,930 | 89,800 | 4.69 | 1.72 |
| Virgibacillus sp. SK37 | 189,815 | 1,017 | 4.97 | 0.03 |
| Bac48 | 225,344 | 108,300 | 5.05 | 2.43 |
| Bac324 | 210,858 | 149,500 | 5.19 | 3.68 |
| Bac144 | 243,038 | 28,200 | 5.29 | 0.61 |
| Bacillus halodurans C-125 | 240,785 | 57,900 | 5.73 | 1.38 |
| Bac84 | 252,616 | 139,900 | 5.77 | 3.20 |
| Bac111 | 244,967 | 35,000 | 6.19 | 0.88 |
| Bacillus licheniformis ATCC 14580 | 282,427 | 214,400 | 6.69 | 5.08 |
| Bacillus subtilis subsp. subtilis str. 168 | 290,389 | 233,200 | 6.89 | 5.53 |
| Bac330 | 309,859 | 138,500 | 6.95 | 3.11 |
| Bacillus clausii KSM-K16 | 314,170 | 92,100 | 7.30 | 2.14 |
| Bac332 | 434,257 | 190,400 | 9.38 | 4.11 |
| Bac57 | 418,960 | 297,600 | 9.91 | 7.04 |
| Bacillus amyloliquefaciens DSM7 | 566,216 | 248,100 | 14.23 | 6.23 |
| Geobacillus kaustophilus HTA426 DNA | 690,645 | 52,600 | 19.48 | 1.48 |

**Supplementary Table S3**. Reactions that are enriched in at least 75% of the metabolic networks of Red Sea species and are absent in at least 90% of the other networks

| **id** | **name** |
| --- | --- |
| TRANS-RXN0-204 | Transport of D-glucarate |
| 3.6.3.24-RXN | no name 3.6.3.24-RXN |
| TRANS-RXN-98 | transporrt of a glucuronide |
| TRANS-RXN0-203 | Transport of D-galactarate |
| RXN-18016 | teichoic acid poly(ribitol phosphate) polymerase |
| 4.2.2.2-RXN | pectate lyase |
| RXN-16245 | 4-methyl-2-oxopentanoate reductase |
| 3.2.1.120-RXN | oligoxyloglucan beta-glycosidase |
| RXN-11720 | chloroethylmalonyl-CoA decarboxylase |
| TRANS-RXN0-523 | Transport of D-glycerate |
| CATECHOL-23-DIOXYGENASE-RXN | catechol dioxygenase |
| 2.3.1.41-RXN | beta-ketoacyl [acyl-carrier protein] synthase |
| LACTALDDEHYDROG-RXN | NAD+ L-lactaldehyde dehydrogenase |
| RXN-17076 | 3-hydroxy-4-methyl-anthranilate pentapeptide lactone synthase |
| RXN0-3281 | formate dehydrogenase |
| RHAMNULOKIN-RXN | rhamnulokinase |
| RXN-18027 | polyisoprenyl-teichoic acid--peptidoglycan teichoic acid transferase |

**Supplementary Table S4.** Normalized position of cluster in genome, where cluster is detected

| **cluster id** | **Bac_111** | **Bac_144** | **Bac_324** | **Bac_330** | **Bac_332** | **Bac_44** | **Bac_48** | **Bac_57** | **Bac_84** | **Bac_94** | **DSM7** | **str_Ames** | **str_Ames_Ancestor** | **str_Sterne** | **ATCC_10987** | **ATCC_14579** | **E33L** | **KSM_K16** | **C_125** | **ATCC_14580** | **P2366** | **SAFR_032** | **str_168** | **konkukian_str** | **Al_Hakam** | **KBAB4** | **HTA426** | **HTE831** | **SK37** |
| --- | --- | --- | --- | --- | --- | --- | --- | --- | --- | --- | --- | --- | --- | --- | --- | --- | --- | --- | --- | --- | --- | --- | --- | --- | --- | --- | --- | --- | --- |
| **cluster1** | 0.758345476 |  |  |  | 0.367943088 |  | 0.897583786 | 0.600245844 | 0.886474712 |  | 0.634902174 | 0.422380762 |  | 0.422290364 | 0.43850448 |  | 0.425623407 |  |  | 0.881889984 |  |  | 0.77944713 | 0.425578911 | 0.430614288 | 0.425963964 |  |  |  |
| **cluster2** | 0.309223716 |  |  |  |  | 0.184386714 | 0.310944115 | 0.264187992 | 0.292654206 |  | 0.309838151 |  |  |  |  |  |  |  |  | 0.292049182 |  | 0.310055279 | 0.287152784 |  |  |  |  |  |  |
| **cluster3** |  |  |  |  |  |  |  |  |  |  |  | 0.227385953 | 0.226178636 | 0.226109332 | 0.256378244 | 0.219390041 | 0.228950191 | 0.893301983 |  |  |  |  |  | 0.231139176 | 0.232356354 | 0.226486407 |  |  |  |
| **cluster4** | 0.849061432 |  |  |  |  | 0.871817098 | 0.871226717 | 0.853829518 | 0.860440922 |  | 0.839725099 |  |  |  |  |  |  |  |  | 0.857640926 |  | 0.858566188 | 0.866788547 |  |  |  |  |  |  |
| **cluster5** |  |  |  |  |  |  |  |  |  |  |  | 0.096052967 | 0.096050651 | 0.096033441 | 0.109840336 | 0.088512732 | 0.091275468 |  |  |  |  |  |  | 0.09367369 | 0.095855579 | 0.092178556 |  |  |  |
| **cluster6** |  |  |  |  |  |  |  |  |  |  |  | 0.219335897 | 0.21933061 | 0.219257772 | 0.24955147 | 0.212746791 | 0.222211071 |  |  |  |  |  |  | 0.223369899 | 0.225560296 | 0.219662535 |  |  |  |
| **cluster7** |  |  |  |  |  |  |  |  |  |  |  | 0.23359232 | 0.233610028 | 0.23354412 | 0.263658477 | 0.226567771 | 0.235900123 |  |  |  |  |  |  | 0.237231184 | 0.239687405 | 0.233954235 |  |  |  |
| **cluster8** |  |  |  |  |  |  |  |  |  |  |  | 0.477295227 | 0.477307444 | 0.47717887 | 0.49415537 | 0.493501434 | 0.483643013 |  |  |  |  |  |  | 0.486526673 | 0.491500908 | 0.482567562 |  |  |  |
| **cluster9** |  |  |  |  |  |  |  |  |  |  |  | 0.635478535 | 0.635487513 | 0.635444759 | 0.636900126 | 0.652115125 | 0.640292761 |  |  |  |  |  |  | 0.647290061 | 0.644725096 | 0.629452618 |  |  |  |
| **cluster10** |  |  |  |  |  |  |  |  |  |  |  | 0.987594057 | 0.987594356 | 0.987597499 | 0.983831753 | 0.987537809 | 0.98740076 |  |  |  |  |  |  | 0.987000643 | 0.987671984 | 0.986839548 |  |  |  |
| **cluster11** | 0.516256936 |  |  |  |  |  | 0.531240613 | 0.511259272 | 0.517042011 |  | 0.509579169 |  |  |  |  |  |  |  |  | 0.523489099 |  | 0.517907943 | 0.498888416 |  |  |  |  |  |  |
| **cluster12** | 0.536366718 |  |  |  |  |  | 0.549371906 | 0.52976504 | 0.535534385 |  | 0.528666406 |  |  |  |  |  |  |  |  | 0.542934715 |  | 0.537922568 | 0.549744094 |  |  |  |  |  |  |
| **cluster13** | 0.818698445 |  |  |  |  |  | 0.846921443 | 0.819787518 | 0.83600155 |  | 0.77356999 |  |  |  |  |  |  |  |  | 0.832321081 |  | 0.825589578 | 0.83573026 |  |  |  |  |  |  |
| **cluster14** |  |  |  |  |  |  |  |  |  |  |  | 0.327238879 | 0.327254521 | 0.327166142 | 0.353741461 | 0.316445296 | 0.327779544 |  |  |  |  |  |  | 0.334018274 | 0.332860607 |  |  |  |  |
| **cluster15** |  |  |  |  |  |  |  |  |  |  |  | 0.357407457 | 0.357422372 | 0.357326624 |  | 0.35654806 | 0.363096937 |  |  |  |  |  |  | 0.363791177 | 0.364129706 | 0.364313504 |  |  |  |
| **cluster16** |  |  |  |  |  |  |  |  |  |  |  | 0.572457197 | 0.572467885 | 0.572056183 |  |  | 0.57998355 |  |  |  |  |  |  | 0.589772632 | 0.582705245 | 0.572362964 |  |  |  |
| **cluster17** | 0.283841564 |  |  |  |  |  | 0.278080321 | 0.242211062 | 0.260449855 |  | 0.285245411 |  |  |  |  |  |  |  |  | 0.26036335 |  |  | 0.259336024 |  |  |  |  |  |  |
| **cluster18** |  |  | 0.588800153 | 0.374162258 | 0.621764372 |  |  |  |  |  |  |  |  |  |  |  |  | 0.085122788 | 0.236354308 |  |  |  |  |  |  |  |  | 0.151926937 | 0.632052271 |
| **cluster19** |  |  |  |  |  |  |  |  |  |  |  | 0.533394053 | 0.533405683 | 0.533343419 |  | 0.547473128 | 0.53941395 |  |  |  |  |  |  | 0.541070267 | 0.545929108 |  |  |  |  |
| **cluster20** | 0.488983097 |  |  |  |  |  | 0.497493941 | 0.511259272 | 0.48296096 |  | 0.509579169 |  |  |  |  |  |  |  |  |  |  |  | 0.468632268 |  |  |  |  |  |  |
| **cluster21** |  | 0.348387145 |  |  |  |  |  |  |  |  |  |  |  |  |  |  |  |  |  |  |  | 0.296133552 |  |  |  |  |  |  |  |
| **cluster22** | 0.907014929 |  |  |  |  |  |  | 0.866332091 |  |  | 0.868272918 |  |  |  |  |  |  |  |  |  |  | 0.915886317 | 0.91821591 |  |  |  |  |  |  |
| **cluster23** |  |  |  |  |  |  |  |  |  |  |  | 0.347688852 | 0.347704001 | 0.347610565 |  |  | 0.353352865 |  |  |  |  |  |  |  | 0.35448226 |  |  |  |  |
| **cluster24** |  |  |  |  |  |  |  |  |  |  |  | 0.628122242 | 0.628131397 |  |  |  | 0.632847914 |  |  |  |  |  |  | 0.639725073 | 0.637304357 |  |  |  |  |
| **cluster25** | 0.101104697 |  |  |  |  |  |  | 0.082761786 |  |  | 0.08773192 |  |  |  |  |  |  |  |  |  |  |  | 0.093205461 |  |  |  |  |  |  |
| **cluster26** | 0.298333886 |  |  |  |  |  |  | 0.25510417 |  |  | 0.299649214 |  |  |  |  |  |  |  |  |  |  |  | 0.27524726 |  |  |  |  |  |  |
| **cluster27** |  |  |  |  |  |  | 0.095725141 |  | 0.091079939 |  |  |  |  |  |  |  |  |  |  | 0.092276388 |  | 0.098214645 |  |  |  |  |  |  |  |
| **cluster28** | 0.762806275 |  |  |  |  |  |  | 0.770796687 |  |  | 0.769093078 |  |  |  |  |  |  |  |  |  |  |  |  |  |  |  |  |  |  |
| **cluster29** |  |  | 0.48953993 |  | 0.528264697 |  |  |  |  |  |  |  |  |  |  |  |  |  |  |  |  |  |  |  |  |  |  |  | 0.528860865 |
| **cluster30** |  |  | 0.232135148 |  | 0.655733153 |  |  |  |  |  |  |  |  |  |  |  |  |  |  |  |  |  |  |  |  |  |  |  | 0.270321773 |
| **cluster31** |  |  |  |  |  |  | 0.302003346 |  | 0.283486957 |  |  |  |  |  |  |  |  |  |  | 0.283206401 |  |  |  |  |  |  |  |  |  |
| **cluster32** |  |  |  |  |  |  | 0.268213779 |  | 0.24810268 |  |  |  |  |  |  |  |  |  |  | 0.247552276 |  |  |  |  |  |  |  |  |  |
| **cluster33** |  |  |  |  |  |  | 0.217799287 |  | 0.196065944 |  |  |  |  |  |  |  |  |  |  | 0.195483609 |  |  |  |  |  |  |  |  |  |
| **cluster34** |  |  |  |  |  |  | 0.92802216 |  | 0.917598486 |  |  |  |  |  |  |  |  |  |  | 0.914332696 |  |  |  |  |  |  |  |  |  |
| **cluster35** |  |  |  |  |  |  | 0.832587989 |  | 0.821292049 |  |  |  |  |  |  |  |  |  |  | 0.80916365 |  |  |  |  |  |  |  |  |  |
| **cluster36** |  |  |  |  |  |  |  | 0.171956344 |  |  | 0.182785836 |  |  |  |  |  |  |  |  |  |  | 0.205783907 |  |  |  |  |  |  |  |
| **cluster37** |  |  |  |  |  |  |  | 0.427234783 |  |  | 0.457655007 |  |  |  |  |  |  |  |  |  |  |  | 0.432025194 |  |  |  |  |  |  |
| **cluster38** |  |  |  |  |  |  |  |  |  |  |  | 0.957911772 | 0.957912786 |  |  |  | 0.956928662 |  |  |  |  |  |  |  |  |  |  |  |  |
| **cluster39** | 0.189273393 |  |  |  |  |  |  |  |  |  |  |  |  |  |  |  |  |  |  |  |  | 0.186556268 |  |  |  |  |  |  |  |
| **cluster40** | 0.836290147 |  |  |  |  |  |  |  |  |  |  |  |  |  |  |  |  |  |  |  |  |  | 0.854964624 |  |  |  |  |  |  |
| **cluster41** |  | 0.955588534 |  |  |  |  |  |  |  | 0.667851484 |  |  |  |  |  |  |  |  |  |  |  |  |  |  |  |  |  |  |  |
| **cluster42** |  | 0.957809913 |  |  |  |  |  |  |  | 0.670469145 |  |  |  |  |  |  |  |  |  |  |  |  |  |  |  |  |  |  |  |
| **cluster43** |  | 0.341782901 |  |  |  |  |  |  |  |  |  |  |  |  |  |  |  |  |  |  |  |  |  |  |  |  |  |  |  |
| **cluster44** |  |  | 0.067779474 |  |  |  |  |  |  |  |  |  |  |  |  |  |  |  |  |  |  |  |  |  |  |  |  |  | 0.103114465 |
| **cluster45** |  |  |  | 0.83250339 | 0.87999665 |  |  |  |  |  |  |  |  |  |  |  |  |  |  |  |  |  |  |  |  |  |  |  |  |
| **cluster46** |  |  |  |  |  | 0.06968429 |  |  |  |  |  |  |  |  |  |  |  |  |  |  |  |  |  |  |  | 0.653543045 |  |  |  |
| **cluster47** |  |  |  |  |  | 0.661935008 |  |  |  |  |  |  |  |  |  |  |  |  |  |  | 0.428898224 |  |  |  |  |  |  |  |  |
| **cluster48** |  |  |  |  |  |  | 0.592897873 |  | 0.57971818 |  |  |  |  |  |  |  |  |  |  |  |  |  |  |  |  |  |  |  |  |
| **cluster49** |  |  |  |  |  |  |  | 0.980843774 |  |  | 0.91870708 |  |  |  |  |  |  |  |  |  |  |  |  |  |  |  |  |  |  |
| **cluster50** |  |  |  |  |  |  |  | 0.133796273 |  |  | 0.13470118 |  |  |  |  |  |  |  |  |  |  |  |  |  |  |  |  |  |  |
| **cluster51** |  |  |  |  |  |  |  | 0.228210825 |  |  | 0.270581697 |  |  |  |  |  |  |  |  |  |  |  |  |  |  |  |  |  |  |
| **cluster52** |  |  |  |  |  |  |  | 0.498429814 |  |  | 0.4994293 |  |  |  |  |  |  |  |  |  |  |  |  |  |  |  |  |  |  |
| **cluster53** |  |  |  |  |  |  |  |  |  |  |  | 0.612615937 |  |  |  |  |  |  |  |  |  |  |  | 0.624995084 |  |  |  |  |  |
| **cluster54** |  |  |  |  |  |  |  |  |  |  |  |  | 0.306095991 | 0.306013985 |  |  |  |  |  |  |  |  |  |  |  |  |  |  |  |
| **cluster55** |  |  |  |  |  |  |  |  |  |  |  |  | 0.485619098 | 0.485528997 |  |  |  |  |  |  |  |  |  |  |  |  |  |  |  |
